# Types of household headship and associated life satisfaction among older adults in India: findings from LASI survey, 2017–18

**DOI:** 10.1186/s12877-022-02772-7

**Published:** 2022-01-25

**Authors:** Shobhit Srivastava, T. Muhammad, K. M. Sulaiman, Manish Kumar, S. K. Singh

**Affiliations:** 1 Department of Survey Research & Data Analytics, International Institute for Population Sciences, Mumbai, Maharashtra 400088 India; 2 International Institute for Population Sciences, Mumbai, 400088 India

**Keywords:** Household headship, Life satisfaction, Older adults, India

## Abstract

**Background:**

Household headship with decision-making power may have a positive influence on life satisfaction in older adults. This study examines the associations of several types of household headship with life satisfaction among older adults in India.

**Method:**

The study utilized the data from the Longitudinal Ageing Study in India (2017–18). The effective sample size for the study was 31,464 adults aged 60 years or older. Ordered logistic regression model was employed to find the association of life satisfaction with household headship status after adjusting for selected socioeconomic and demographic factors.

**Results:**

It was found that about 1.3% of older male and 1.5% of older females had nominal headship status in their household. Higher percentage of older males (42%) and females (48.3%) who had nominal headship status had low life satisfaction. In multivariable analysis, older adults who practiced nominal headship had significantly higher odds of low life satisfaction in reference to older adults who practiced functional headship [Adjusted odds ratio (AOR): 1.87; confidence interval (CI): 1.45,2.42]. Interaction model reveals that older men who practised nominal headship had significantly higher odds of low life satisfaction in reference to older men who practised functional headship [AOR: 2.34; CI: 1.59,3.45]. Similarly, older women who practised nominal headship had 55% significantly higher likelihood to have low life satisfaction in reference to older men who practised functional headship [AOR: 1.55; CI: 1.09, 2.18].

**Conclusion:**

The recognition of older individuals as active agents of the households they belong to, and giving them the value they deserve may help boosting their mental well-being. As a direct driver of subjective well-being, headship status and decision making power deserve a more prominent role and future studies are required on the mechanisms of functional and nominal headship statuses that have impact on successful aging.

**Supplementary Information:**

The online version contains supplementary material available at 10.1186/s12877-022-02772-7.

## Background

The principal concept in sociology is that older adults will become more likely to live autonomously as societies modernize and develop [1, 2]. Still, in most countries, the family provides support to older adults. Family is a helpful resource to older individuals in their later ages in providing caregiving and giving a sense of merit, lasting emotional bonds, and personal dignity [3, 4]. Inside a family, the power to allocate and control the resources (both economic and social) vested in the head of the household [5], and being the head of the household gives more independence and authority in older ages to adjust with life transitions [6]. Older adults try to maintain their autonomy by holding the headship of their households rather than relocating to with their children or relatives [4, 7]. In developing countries, several societies have rooted in the norms about respect for older adults, and youngsters consider them as their head until they die [2, 8]. Recent studies show a declining status of older adults due to urbanization, industrialization, and Westernization in South Asia [9, 10].

Research shows the effect of ageing on older adults’ capabilities to contribute to their household and society through essential activities [11, 12], and these activities help them evaluate themselves [13]. A cognitive judgmental global evaluation of one’s life is called life satisfaction, and it is not a direct measure of emotion but influence it [14]. Life satisfaction considered an essential aspect of successful ageing and typically conceptualized as one of the crucial aspects of subjective well-being [14, 15]. In recent years, life satisfaction has been estimated and analysed systematically. Self-report questions on life satisfaction are generally incorporated in studies and large scale surveys [13]. In most cases, the older adults recheck their past life and feel satisfied if their goals and dreams are met [16], and this is influenced by the economic and cultural living conditions of older adults [17]. Besides, declining physical health and social support, diminishing cognitive abilities, and lack of economic assistance are associated with ageing, affecting well-being and life satisfaction among older adults [18, 19].

Social networks and support continued significantly correlated with positive mental well-being among older adults and protect the depressive symptoms and their potential from weakening cognition, directly affecting life satisfaction [20, 21]. Functioning independently, particularly in personal care and household activities, means autonomy for older adults, and it has a direct reflection on their life satisfaction [22, 23]. Loneliness and feeling worthless in older ages can result in low life satisfaction [24], To avoid this situation and improve older adults’ life satisfaction, their self-determination and wishes to be respected, and their opinion and decisions are considered in household decision making [22]. In a household, older adults considered to help resolve inter-and intra-family disputes, and as patrons of perceived traditional customs, they are expected to deliver their experiences to younger generations [25, 26]; this role earns them respect [27]. Family-based expected availability of future care and respect significant predictor of the older adult’s life satisfaction [28]. Globalization and modernization facilitated the shattering of social changes, replacing the extended family with nuclear families considered as the main element of older adults losing power in their household [29, 30].

Most of the Indian older adults lives with family, common throughout India [31], and it provides social security, care and economic support to older adults [32, 33]. In the Indian scenario, social and familial support systems are necessary for assuring mental well-being among the ageing population [34]. In India, older adults are treated with honour for their age and wisdom [35], but once the older adults reached a particular age, the household headship is transferred to the next eldest member of the family [36]. This household system supports the older adults in strongly negotiating life’s difficulties in financial and non-financial support [37, 38]. Negotiating on making choices leads to asserting power [39]; in this context, the influence of older adults in decision making reflects the power [40]. Older males are in a relatively better position in asserting power than older women [41]. The traditional Indian society considers that the older adults’ decisions are final [42]; whereas, any restrictions on decision-making power have an adverse effect on their mental well-being [43]. On the other hand, the shift from the joint to the nuclear family, and changing the socio-cultural values impose a threat to older adult’s intra-household decision-making power, which increases the chances of loneliness and lower life satisfaction [44, 45].

The recent evidence points out that the household headship and decision-making power may have a positive influence on subjective wellbeing in older adults [46]. This study intends to examine the associations of being household head with and without decision making power and being not a head with and without decision making power with life satisfaction among older adults in India. Further, this paper examined the association of socio-economic characteristics with older adults’ subjective well-being in India. Based on previous research, this paper hypothesizes that an older adult who is household head with decision-making power is more likely to have better life satisfaction than those who do not have influence in household decision making.

## Methods

### Data

This study utilizes data from India’s first nationally representative longitudinal Ageing survey (LASI-2017-18) which investigates into the health, economics and social determinants and consequences of population ageing in India [47]. The representative sample included 72,250 individuals aged 45 and above and their spouses across all states and union territories of India except Sikkim. The LASI adopts a multistage stratified area probability cluster sampling design to select the eventual units of observation. This study provides scientific evidence on demographics, household economic status, chronic health conditions, symptom-based health condition, functional and mental health, biomarkers, health care utilization, work and employment etc. It enables the cross-state analyses and the cross-national analyses of ageing, health, economic status and social behaviours and has been designed to evaluate the effect of changing policies and behavioural outcomes in India. Detailed information on the sampling frame is available on the LASI wave-1 report [47]. The effective sample size for the present study was 31,464 older adults aged 60 years and above. There were 653 missing cases in some of the variable which were excluded during the adjusted multivariate analysis.

### Variable description

#### Outcome variable

Life satisfaction among older adults was assessed using the questions a. In most ways my life is close to ideal; b. The conditions of my life are excellent; c. I am satisfied with my life d. So far, I have got the important things I want in life; e. If I could live my life again, I would change almost nothing. The responses were categorized as strongly disagree, somewhat disagree, slightly disagree, neither agree nor disagree, slightly agree, somewhat agree and strongly agree. Using the responses to the five statements regarding life satisfaction, a scale was constructed with a score ranging from 5 to 35 with higher score indicating greater life satisfaction. The scale is further categorized into tertiles, that are ‘low satisfaction’ (score of 5–20), ‘medium satisfaction’ (score of 21–25), and ‘high satisfaction’ (score of 26–35) [47]. The categorization was based on existing literature [48]. The outcome variable was coded as 0 “high”, 1 “medium” and 2 “low” (Cronabach alpha: 0.89).

#### Main exposure variable

The main explanatory variable was headship status among older adults i.e. whether the status was nominal, functional, not head but takes decision and nor head neither take decision. The nominal headship was defined as the head that does not have any decision-making power in the household whereas the functional head was the head that has the absolute/partial power to make household decisions. The variable was generated using two variables i.e. first whether the older adult is the head of the household or not and whether he makes the major household decision or not. The sample only includes the older adults who were the heads of the household. The decision-making power was assessed using six questions which include “Who usually makes the following decisions: you alone or with your spouse, with your children, or with others?” on the following issues (a). Marriage of son/daughter. (b). Buying and selling of property (c). Gifts to daughters, grandchildren, other relatives (d). Education of children, grandchildren (e). Arrangement of social and religious events (Cronabach's alpha: 0.89). The responses were coded as 0 “no role in decision making” and 1 “full/partial role in decision making” i.e. decide alone or with your spouse, with your children, or with others. Headship status was coded as 0 “nominal head” which combines head with no role as decision-maker in the household, 1 “functional head” which combines head with full/partial role in decision-maker in the household, 2 “not head but takes decisions” and 3 “not head neither take any decision”.

#### Other exposure variables

Age was categorized as young old (60–69 years), old-old (70–79 years) and oldest old (80+ years). Educational status was categorized as no education/primary not completed, primary, secondary and higher. Living arrangement was categorized as living alone, living with spouse and living with others [49]. Marital status was categorized as currently married, widowed and others. Others included separated/divorced/never married. Working status was categorized as currently working, retired and not working. Social participation was categorized as no and yes. Respondents were said to be socially engaged if they participate in the following activities. Eat out of house (Restaurant/Hotel); Go to park/beach for relaxing/entertainment; Play cards or indoor games; Play outdoor games/sports/exercise/jog/yoga; Visit relatives /friends; Attend cultural performances /shows/Cinema; Attend religious functions /events such as bhajan/satsang/prayer; Attend political/community/organization group meetings; and use a computer for e-mail/net surfing etc.

Self-rated health was coded as good which includes excellent, very good and good where as poor includes fair and poor [50]. Difficulty in ADL (Activities of Daily Living) was coded as no and yes. Activities of Daily Living (ADL) is a term used to refer to normal daily self-care activities (such as movement in bed, changing position from sitting to standing, feeding, bathing, dressing, grooming, personal hygiene etc.) The ability or inability to perform ADLs is used to measure a person’s functional status, especially in the case of people with disabilities and the older adults [51, 52]. Difficulty in IADL (Instrumental Activities of Daily Living) was coded as no and yes. Instrumental activities of daily living that are not necessarily related to the fundamental functioning of a person, but they let an individual live independently in a community. The set ask were necessary for independent functioning in the community. Respondents were asked if they were having any difficulties that were expected to last more than 3 months, such as preparing a hot meal, shopping for groceries, making a telephone call, taking medications, doing work around the house or garden, managing money (such as paying bills and keeping track of expenses), and getting around or finding an address in unfamiliar places [51, 52]. Activities of daily living that are not necessarily related to fundamental functioning of a person, but they let an individual live independently in a community. The set ask were necessary for independent functioning in the community. Respondents were asked if they were having any difficulties that were expected to last more than 3 months, such as preparing a hot meal, shopping for groceries, making a telephone call, taking medications, doing work around the house or garden, managing money, and getting around or finding an address in unfamiliar place s[51, 52]. Psychological distress was coded as low, medium and high. Psychological distress was measured using the following questions a. How often did you have trouble concentrating? b. How often did you feel depressed? c. How often did you feel tired or low in energy? d. How often were you afraid of something? e. How often did you feel you were overall satisfied? f. How often did you feel alone? g. How often were you bothered by things that don’t usually bother you? h. How often did you feel that everything you did was an effort? i. How often did you feel hopeful about the future? j. How often did you feel happy? The response was coded as 1. Rarely or never 2. Sometimes 3. Often and 4. Most or all of the time. The response was coded as per the question in binary form 0 “Rarely or never/ Sometimes” and 1 “Often/ Most or all of the time” (Cronabach's alpha: 0.70) [53].

The monthly per-capita consumption expenditure (MPCE) quintile was assessed using household consumption data. The MPCE is computed and used as the summary measure of consumption. The variable was divided into five quintiles i.e., from poorest to richest [47]. Religion was coded as Hindu, Muslim, Christian, and Others. Caste was recoded as Scheduled Tribe, Scheduled Caste, Other Backward Class, and others [51]. The Scheduled Castes (SCs) and Scheduled Tribes (STs) are among the most disadvantaged socio-economic groups in India. The OBC is the group of people who were identified as “educationally, economically and socially backward”. The OBCs are considered low in the traditional caste hierarchy. The “other” caste category is identified as having higher social status [54]. Place of residence was categorized as rural and urban. The region was coded as North, Central, East, Northeast, West, and South.

### Statistical analysis

Since the outcome variable, life satisfaction, is ordinal, with three categories – “low,” medium,” and “high,” and when the order of the values in a variable is considered, ordered logistic regression is the most commonly used model [55]. Thus, we have employed an ordered logistic regression model to study the association of life satisfaction with household headship status after adjusting for selected socioeconomic and demographic factors. A standard ordered logit model [55] is derived by defining a latent variable z, which models the ordinal ranking of the data. It is assumed that the discrete life satisfaction levels are associated with this continuous latent variable. This latent variable is generally specified as a linear function as follows:

1 $$z=\beta {X}_i+{\in}_i, for\;i= 1, 2,\dots \dots \dots \dots \dots, N$$

Where *i* (*i = 1, 2,……………, N)* represents the individuals*,* Xi is a vector of independent variables (excluding a constant), β is a vector of unknown parameters to be estimated, and *ϵ*_*i*_ is a random disturbance term. By using the Eq. (1), the observed life satisfaction variable (y), which is ordinal, for each observation can be defined as:

2 $${\displaystyle \begin{array}{c}y=1\kern0.24em if\;z\le {\mu}_0(Low)\\ {}y=2\kern0.24em if{\mu}_0\le z\le {\mu}_1(Medium)\\ {}y=3\kern0.24em if\;{\mu}_1\le z\le {\mu}_2(High)\end{array}}$$

Where; *μ*_*i*_ are the unknown parameters to be estimated (also referred to as thresholds) corresponds to integer ordering. y. in order to assure a well-defined intervals and the natural ordering of the severity level, the thresholds are assumed to be in the ascending order, such that *μ*_1_ < *μ*_2_ < *μ*_3_, where, *μ*_0_ = −∞ and *μ*_2_ = +∞. To estimate the parameters μ_i_ with the model parameters *β*, an assumption is made on the distribution of *ϵ*_*i*_. If the random error term is assumed to be independent and identically distributed with the logistic distribution, an ordered logit model is derived. However, an ordered probit model would be used if the random error terms are assumed to be normally distributed across observations. The probability that an individual belongs to either of the three categories is defined as:

3 $${\displaystyle \begin{array}{c}P\left(\mathrm{y}=1\right)=\varLambda \left(-\beta \mathrm{X}\right)\\ {}P\left(\mathrm{y}=2\right)=\varLambda \left({\mu}_1-\beta \mathrm{X}\right)-\varLambda \left(-\beta \mathrm{X}\right)\\ {}P\left(\mathrm{y}=3\right)=\varLambda \left({\mu}_2-\beta \mathrm{X}\right)-\varLambda \left({\mu}_1-\beta X\right)\end{array}}$$

Where, *Λ*(∙) is defined as the standard logistic cumulative distribution function. The probability expressions are given as

4 $$\mathrm{P}\left(y=j\right)=\varLambda \left({\mu}_j-\beta \mathrm{X}\right)-\varLambda \left({\mu}_{j-1}-\beta \mathrm{X}\right)$$

Where, *μ*_*j*_ and *μ*_*j* + 1_ represents the upper and lower thresholds for the outcome *j*. The log-likelihood estimation is used to calculate the parameters estimates. For the population of N observations, the likelihood function for the ordered logistic model is given as:

5 $$\mathrm{LL}={\sum}_{i=1}^N{\sum}_{j=1}^l{\delta}_{ji}\mathit{\ln}\left[\varLambda \left({\mu}_j\hbox{-} {X}_i\beta \right)\hbox{-} \varLambda \left({\mu}_{j-1}\hbox{-} {X}_i\beta \right)\right]$$

Where, *δ*_*ji*_ = 1 if the observed discrete outcome is i, and 0 if it is not. The odds are estimated as the exponents of the coefficient of the parameter of interest and are interpreted as the probability of an event over the probability the event does not occur. The odds of a life satisfaction outcome i is given as:

6 $$\frac{P\left(y=i\right)}{1-P\left(y=i\right)}=\exp \left({\beta}_0+{\beta}_1\mathrm{X}\right)={e}^{\beta_0}{\left({e}^{\beta_1}\right)}^{\mathrm{X}}$$

The multicollinearity among the variables was checked using variance inflation factor (VIF) [56], and found no evidence of multicollinearity. The complex survey design effects were adjusted by using STATA *svyset* and *svy* commands. The whole statistical analyses were performed by using STATA version 14 [57].

## Results

Table 1 revealed socio-economic profile of older adults in India. Figure-S1 reveals the percentage of older adults involved in different types of decision making in the family. It was found that about 1.3% of older male and 1.5% of older females had nominal headship status in their household. However, about 85.8% of older males practiced functional headship and only 22.4% of older females practised functional headship. About 53.1% of older males and 81.4% of older females were not educated or did not completed their primary schooling. About 2.5 and 8.5% of older male and older females were living alone respectively. Nearly, 16.5 and 54% of older male and females were widowed respectively. Almost, 43.8 and 19% of older males and females were working respectively. Only, 11.2 and 15.0% of older males and females had social participation. About 46.7 and 50.2% of older male and females had poor self-rated health. Nearly, 21.9 and 26.5% of older male and females had difficulty in ADL respectively. About 39.7 and 56.9% of older male and females had difficulty in IADL respectively. About 26.2 and 31.4% of older males and females had low psychological distress respectively.

**Table 1:** Socio-economic profile of older adults in India, 2017–18

Background characteristics	Male		Female	
	Sample	Percentage	Sample	Percentage
**Headship status**^**a**^				
Nominal head	145	1.3	189	1.5
Functional head	9611	85.8	2773	22.4
Not head but take decision	1310	11.7	8807	71.0
Nor head neither take any decision	132	1.2	633	5.1
**Age**				
Young-old	8730	57.8	9678	59.1
Old-old	4702	31.1	4803	29.4
Oldest-old	1666	11.0	1886	11.5
**Education**				
No education/primary not completed	8019	53.1	13,314	81.4
Primary completed	2235	14.8	1297	7.9
Secondary completed	3096	20.5	1297	7.9
Higher and above	1748	11.6	458	2.8
**Living arrangement**				
Living alone	380	2.5	1397	8.5
Living with spouse	3929	26.0	2485	15.2
Living with children	10,205	67.6	11,268	68.9
Living with others	583	3.9	1216	7.4
**Marital status**				
Currently married	12,242	81.1	7211	44.1
Widowed	2489	16.5	8837	54.0
Others	366	2.4	318	2.0
**Working status**				
Working	6613	43.8	3108	19.0
Retired	7907	52.4	5593	34.2
Not working	578	3.8	7665	46.8
**Social participation**				
No	13,409	88.8	13,914	85.0
Yes	1689	11.2	2452	15.0
**Self-rated health**^**a**^				
Good	7875	53.3	7982	49.8
Poor	6909	46.7	8045	50.2
**Difficulty in ADL**				
No	11,788	78.1	12,022	73.5
Yes	3310	21.9	4344	26.5
**Difficulty in IADL**				
No	9112	60.4	7047	43.1
Yes	5986	39.7	9319	56.9
**Psychological distress**				
Low	6180	40.9	5962	36.4
Medium	4956	32.8	5261	32.2
High	3962	26.2	5143	31.4
**MPCE quintile**				
Poorest	3145	20.8	3681	22.5
Poorer	3219	21.3	3611	22.1
Middle	3262	21.6	3331	20.4
Richer	2902	19.2	3136	19.2
Richest	2570	17.0	2607	15.9
**Religion**				
Hindu	12,386	82.0	13,484	82.4
Muslim	1769	11.7	1781	10.9
Christian	388	2.6	511	3.1
Others	555	3.7	590	3.6
**Caste**				
Scheduled Caste	2836	18.8	3113	19.0
Scheduled Tribe	1166	7.7	1389	8.5
Other Backward Class	6925	45.9	7308	44.7
Others	4172	27.6	4556	27.8
**Place of residence**				
Rural	10,879	72.1	11,322	69.2
Urban	4219	28.0	5044	30.8
**Region**				
North	1863	12.3	2096	12.8
Central	3395	22.5	3202	19.6
East	3713	24.6	3729	22.8
Northeast	437	2.9	497	3.0
West	2457	16.3	2941	18.0
South	3233	21.4	3900	23.8
**Total**	15,098	100.0	16,366	100.0

*MPCE* Monthly per capita consumption expenditure, *ADL* Activities of daily living, *IADL* Instrumental activities of daily living

^a^Sample size may differ because of missing cases/respondent non-response

Table 2 represents percentage of older adults with the degree of life satisfaction by their background characteristics in India. Higher percentage of older males (42%) and females (48.3%) who had nominal headship status had low life satisfaction.

**Table 2:** Percentage of older adults with the degree of life satisfaction by their background characteristics in India, 2017–18

Background characteristics	Male			Female		
	High (%)	Medium (%)	Low (%)	High (%)	Medium (%)	Low (%)
**Headship status**						
Nominal head	45.2	12.9	42.0	23.1	28.6	48.3
Functional head	48.3	22.8	29.0	42.5	21.0	36.5
Not head but take decision	42.0	25.2	32.8	47.4	23.2	29.4
Nor head neither take any decision	46.1	19.7	34.2	35.8	17.9	46.3
**Age**						
Young-old	46.1	23.2	30.7	45.0	22.5	32.5
Old-old	49.7	20.8	29.5	42.5	21.7	35.8
Oldest-old	45.8	23.4	30.8	42.3	22.6	35.1
**Education**						
No education/primary not completed	40.0	24.1	35.9	39.9	22.9	37.2
Primary completed	47.4	21.5	31.1	53.9	22.8	23.3
Secondary completed	55.9	21.8	22.2	69.6	14.6	15.9
Higher and above	64.4	17.6	18.0	61.1	24.1	14.8
**Living arrangement**						
Living alone	36.6	17.7	45.7	32.0	19.8	48.3
Living with spouse	46.6	21.5	32.0	43.5	25.4	31.1
Living with children	48.2	23.0	28.8	46.4	22.1	31.5
Living with others	39.0	23.4	37.7	35.5	20.2	44.3
**Marital status**						
Currently married	47.7	22.6	29.8	46.9	23.5	29.6
Widowed	47.0	21.7	31.4	41.9	21.3	36.8
Others	31.2	25.9	42.8	34.3	20.1	45.6
**Working status**						
Working	45.7	24.3	30.0	41.6	22.6	35.8
Retired	48.7	20.8	30.4	42.8	22.0	35.2
Not working	43.7	23.3	33.0	45.8	22.3	31.9
**Social participation**						
No	47.7	23.0	29.3	44.6	23.0	32.5
Yes	42.6	18.0	39.4	40.3	18.0	41.7
**Self-rated health**						
Good	52.3	21.8	26.0	49.5	20.9	29.7
Poor	41.3	23.3	35.4	38.6	23.6	37.8
**Difficulty in ADL**						
No	48.4	22.2	29.4	45.7	22.2	32.0
Yes	42.1	23.8	34.2	38.8	22.3	38.9
**Difficulty in IADL**						
No	50.4	21.4	28.2	47.1	22.5	30.4
Yes	41.8	24.3	33.8	41.5	22.1	36.4
**Psychological distress**						
Low	63.7	18.4	17.9	60.7	19.6	19.7
Medium	43.4	25.1	31.5	42.2	23.3	34.6
High	28.8	24.9	46.3	28.2	24.0	47.8
**MPCE quintile**						
Poorest	40.7	23.6	35.8	35.1	24.3	40.5
Poorer	45.5	23.2	31.2	40.7	22.7	36.6
Middle	47.2	24.0	28.8	46.6	21.8	31.6
Richer	50.9	21.9	27.2	48.6	21.9	29.4
Richest	53.0	18.9	28.1	52.0	19.7	28.3
**Religion**						
Hindu	47.3	22.1	30.6	44.1	22.2	33.7
Muslim	46.4	24.4	29.2	40.8	23.4	35.8
Christian	46.6	19.9	33.6	42.9	19.3	37.8
Others	47.4	26.3	26.3	51.8	21.8	26.4
**Caste**						
Scheduled Caste	38.9	23.1	38.1	36.3	23.1	40.6
Scheduled Tribe	41.8	22.9	35.3	38.1	23.2	38.6
Other Backward Class	48.1	22.0	30.0	45.2	21.8	33.1
Others	52.9	22.8	24.3	49.0	22.2	28.8
**Place of residence**						
Rural	44.3	23.5	32.2	40.2	23.4	36.4
Urban	55.0	19.7	25.3	52.6	19.6	27.8
**Region**						
North	42.5	24.8	32.8	41.4	23.9	34.7
Central	43.9	25.3	30.9	39.7	25.8	34.6
East	40.9	26.6	32.4	34.4	25.2	40.4
Northeast	50.8	27.5	21.7	40.4	29.7	30.0
West	71.1	14.0	14.9	66.5	16.4	17.1
South	41.7	19.0	39.3	41.5	19.1	39.4
**Total**	47.2	22.5	30.4	44.0	22.3	33.8

Table 3 represents the logistic regression estimates for life satisfaction among older adults by their background characteristics. There were 653 missing cases in SRH variable therefore the regression model was run on 30,811 cases. Model-1 which represents unadjusted odds revealed that older adults who practiced nominal headship had significantly higher odds to suffer from low life satisfaction in reference to older adults who practiced functional headship [UOR: 2.31; CI: 1.80,2.95]. Even older adults who were not head neither take any decision had significantly higher odds to suffer from low life satisfaction in comparison to older adults who practiced functional headship [UOR: 1.77; CI: 1.51,2.09].

**Table 3:** Logistic regression estimates for life satisfaction among older adults by their background characteristics (*n* = 30,811), 2017–18

Background characteristics	Model-1	Model-2	Model-3
	UOR (95% CI)	AOR (95% CI)	AOR (95% CI)
**Headship status**			
Nominal head	2.31*(1.80,2.95)	1.87*(1.45,2.42)	
Functional head	Ref.	Ref.	
Not head but take decision	1.00(0.95,1.05)	0.91*(0.85,0.98)	
Nor head neither take any decision	1.77*(1.51,2.09)	1.52*(1.28,1.81)	
**Age**			
Young-old		Ref.	
Old-old		0.93*(0.89,0.98)	
Oldest-old		0.81*(0.75,0.88)	
**Sex**			
Male		Ref.	
Female		0.94*(0.88,0.99)	
**Education**			
No education/primary not completed		1.95*(1.76,2.15)	
Primary completed		1.60*(1.44,1.79)	
Secondary completed		1.31*(1.18,1.45)	
Higher and above		Ref.	
**Living arrangement**			
Living alone		1.27*(1.1,1.46)	
Living with spouse		0.90(0.80,1.02)	
Living with children		0.86*(0.77,0.95)	
Living with others		Ref.	
**Marital status**			
Currently married		0.9(0.77,1.04)	
widowed		0.93(0.8,1.08)	
Others		Ref.	
**Working status**			
Working		Ref.	
Retired		0.93*(0.88,0.98)	
Not working		0.9*(0.84,0.96)	
**Social participation**			
No		Ref.	
Yes		1.01(0.95,1.08)	
**Self-rated health**			
Good		Ref.	
Poor		1.32*(1.26,1.38)	
**Difficulty in ADL**			
No		Ref.	
Yes		0.98(0.92,1.04)	
**Difficulty in IADL**			
No		Ref.	
Yes		1.13*(1.08,1.19)	
**Psychological distress**			
Low		Ref.	
Medium		1.93*(1.83,2.03)	
High		2.99*(2.83,3.17)	
**MPCE quintile**			
Poorest		1.31*(1.22,1.41)	
Poorer		1.13*(1.05,1.22)	
Middle		1.10*(1.02,1.18)	
Richer		1.06(0.98,1.14)	
Richest		Ref.	
**Religion**			
Hindu		Ref.	
Muslim		1.16*(1.08,1.24)	
Christian		0.93(0.85,1.02)	
Others		0.95(0.85,1.06)	
**Caste**			
Scheduled Caste		1.20*(1.12,1.29)	
Scheduled Tribe		1.18*(1.09,1.28)	
Other Backward Class		0.99(0.93,1.05)	
Others		Ref.	
**Place of residence**			
Rural		1.11*(1.05,1.17)	
Urban		Ref.	
**Region**			
North		Ref.	
Central		1.01(0.93,1.1)	
East		1.35*(1.26,1.46)	
Northeast		1.05(0.96,1.16)	
West		0.50*(0.46,0.54)	
South		1.19*(1.11,1.28)	
**Headship status # sex**			
Functional head # male			Ref.
Nominal head # male			2.30*(1.55,3.45)
Nominal head # female			1.55*(1.09,2.18)
Functional head # female			0.99(0.89,1.10)
Not head but take decision # male			0.93(0.82,1.04)
Not head but take decision # female			0.90*(0.84,0.96)
Nor head neither take any decision # male			1.07(0.69,1.65)
Nor head neither take any decision # female			1.55*(1.29,1.92)
/cut1	0.72	1.21	1.21
/cut2	1.87	2.34	2.34

*Ref* Reference; #: Interaction effect; *UOR* Unadjusted odds ratio, *AOR* Adjusted odds ratio; * if *p* < 0.05; *CI* Confidence interval; Model-3 was adjusted for all the background characteristics

Model-2 revealed adjusted odds and it was found that older adults who practiced nominal headship had significantly higher odds to suffer from low life satisfaction in reference to older adults who practiced functional headship [AOR: 1.87; CI: 1.45,2.42]. Even older adults who were not head neither take any decision had significantly higher odds to suffer from low life satisfaction in comparison to older adults who practiced functional headship [AOR: 1.52; CI: 1.28,1.81]. Model-3 revealed interaction effects (adjusted for all the background characteristics) and it was found that older males who practised nominal headship had significantly higher odds to have low life satisfaction in reference to older men who practised functional headship [AOR: 2.30; CI: 1.55,3.45]. Similarly, older females who practised nominal headship had 55% significantly higher likelihood to have low life satisfaction in reference to older men who practised functional headship [AOR: 1.55; CI: 1.09, 2.18]. Table-S1 in *supplementary file* represents the sensitivity analysis by sex differences.

## Discussion

The study explored the relationship of different types of household headship and life satisfaction in older people in India. Results showed a low life satisfaction among older participants who had no role in household decision making processes which was consistent with a recent study that found a lower subjective well-being among older adults with nominal headship status than functional headship status [46]. A low degree of independence was found to be correlated with lower life satisfaction among the participants in previous studies of non-institutionalized older adults [58, 59]. Several studies in community settings have also shown that increased dependency is related to low life satisfaction in older people [60], and self-perceived decisional power can mediate the effect of functional capacity over life satisfaction [61]. Also, autonomy and decision making power may facilitate the exercise of older individuals’ will and making choices that boost dignity, value for age, and self-respect [62–64].

Boyle in 2005 has found that autonomy in terms of decision-making power that includes being household head can be especially protective against mental disorders and depressive symptoms, but if their adult children no longer take their opinion into account for important decisions, there would be no autonomy associated, and no protection against mental illnesses [65]. Consistently, our findings suggest that household headship without decision making power is associated with lower levels of life satisfaction. This further supports the findings of past research that demonstrated that participation or commitment to a greater number of productive activities would be positively related to subjective well-being [11].

Furthermore, evidence suggests that as a social stereotype in many developing countries, men are expected to inhibit their emotions to avoid being feminine and any losses of control in life results in the decline of mental wellbeing for older men but not for older women [66]. In the present study, interaction analysis shows that older male participants had a stronger association of nominal household headship (headship with no decision making role) with expressing lower life satisfaction. This again confirms the finding that the decision-making has always been associated with men and being household heads, a decline in the role in decision-making may make them more dissatisfied compared to older women [44]. On the other hand, in the case of decision-making power with neither of the headship statuses, women had higher odds of low life satisfaction associated with no decision making power compared to their male counterparts. This may be attributed to the reaction of older women to their subordinate roles in the household decision making. This can also be partially explained by the gender differences in reporting health status and wellbeing, for example, older women in general, report more health-related problems and low satisfaction than men [67, 68]. However, since there may be gender bias in decision making power in a household for example, older wives may have significant role in household decision making than their husbands, future research should analyze dyadic/household data to examine the gender balance of decision-making power.

The results of our study are limited by its cross-sectional design and missing cases of some health-related variables in multivariate analyses. A better understanding of the headship status and wellbeing in this study may also be enhanced in future research efforts by attention to some other following issues. First, a wider range of indicators of subjective well-being such as happiness, self-esteem, and self-efficacy should be considered. The questions used to generate decision making power in the study only capture the overall freedom they perceive in deciding about household activities. Also, the decision-making power used in our study captures a general evaluation of older members’ role making in the households and thus gives no information on their perceived power in other life domains such as at work or outside the family. Similarly, there might be adverse effects of older adults’ decision-making power on other family members. For example, when older parents decide the marriage of the children of son or daughter, son/daughter may not always accept or respect such a decision. Thus, in future studies on the effects of decision-making power on life satisfaction, it may be better to approach not only older adults but also families as a whole. Also, the influence of engagement in multiple productive activities and the intensity of that engagement for well-being should be extended to analyses of physical health and mortality.

## Conclusion

The findings of our study suggest that healthy aging is a process or a consequence of the value accorded to or decision making power attributed to older people that enhance their happiness, and life satisfaction. The recognition of older individuals as active agents of the households they belong to and giving them the value they deserve, may help boosting their mental well-being. Despite constituting a pivotal part of late-life mental health, the significance of older individuals’ active involvement in household decision making processes has been undervalued in the investigation of their subjective well-being. As a direct driver of subjective well-being, headship status and decision-making power deserve a more prominent role and future studies are required on the mechanisms of functional and nominal headship statuses that have impact on successful aging.

## Supplementary Information

**Additional file 1:** **Figure S1.** Percentage of older adults involved in different types of decision making in the family. **Table S1.** Logistic regression estimates for life satisfaction among older adults by their background characteristics (*n*=30,811), 2017-18.

## Data Availability

The study uses secondary data which is available on reasonable request through https://www.iipsindia.ac.in/content/lasi-wave-i.

## Abbreviations

AOR: Adjusted odds ratio
CI: Confidence interval
ADL: Activities of daily living
IADL: Instrumental activities of daily living.
MPCE: Monthly per capita consumption expenditure
