# Supplementary material for: Types of household headship and associated life satisfaction among older adults in India: findings from LASI survey, 2017–18
Source: BMC Geriatr. 2022 Jan 25;22:78. doi: 10.1186/s12877-022-02772-7 (PMC8790881; doi:10.1186/s12877-022-02772-7)
Supplement: Supplementary file 1 — Additional file 1: Figure S1. Percentage of older adults involved in different types of decision making in the family. Table S1. Logistic regression estimates for life satisfaction among older adults by their background characteristics (n=30,811), 2017-18. [file 12877_2022_2772_MOESM1_ESM.docx]

**Figure-S1.** Percentage of older adults involved in different types of decision making in the family

| **Table-S1.** Logistic regression estimates for life satisfaction among older adults by their background characteristics (n=30,811), 2017-18 | | |
| --- | --- | --- |
| **Background characteristics** | **Male** | **Female** |
|  | **AOR (95% CI)** | **AOR (95% CI)** |
| **Headship status** |  |  |
| Nominal head | 2.29*(1.56,3.39) | 1.57*(1.12,2.22) |
| Functional head | Ref. | Ref. |
| Not head but take decision | 0.94(0.83,1.07) | 0.89*(0.80,0.98) |
| Not head neither take any decision | 1.10(0.71,1.71) | 1.57*(1.29,1.92) |
| **Age** |  |  |
| Young-old | Ref. | Ref. |
| Old-old | 0.86*(0.8,0.92) | 1.01(0.94,1.08) |
| Oldest-old | 0.78*(0.7,0.88) | 0.85*(0.76,0.94) |
| **Education** |  |  |
| No education/primary not completed | 2.05*(1.82,2.32) | 1.76*(1.45,2.12) |
| Primary completed | 1.7*(1.48,1.94) | 1.45*(1.18,1.79) |
| Secondary completed | 1.39*(1.23,1.57) | 1.15(0.93,1.42) |
| Higher and above | Ref. | Ref. |
| **Living arrangement** |  |  |
| Living alone | 1.41*(1.07,1.85) | 1.22*(1.04,1.43) |
| Living with spouse | 0.81(0.65,1.01) | 0.95(0.81,1.12) |
| Living with children | 0.79*(0.64,0.97) | 0.88*(0.78,1) |
| Living with others | Ref. | Ref. |
| **Marital status** |  |  |
| Currently married | 1.03(0.81,1.3) | 0.82(0.67,1.01) |
| widowed | 0.95(0.76,1.2) | 0.9(0.74,1.1) |
| Others | Ref. | Ref. |
| **Working status** |  |  |
| Working | Ref. | Ref. |
| Retired | 0.93*(0.86,1) | 0.92(0.84,1.01) |
| Not working | 0.98(0.84,1.14) | 0.89*(0.81,0.97) |
| **Social participation** |  |  |
| No | 1.02(0.91,1.13) | 1.02(0.93,1.11) |
| Yes | Ref. | Ref. |
| **Self-rated health** |  |  |
| Good | Ref. | Ref. |
| Poor | 1.33*(1.24,1.42) | 1.31*(1.23,1.39) |
| **Difficulty in ADL** |  |  |
| No | Ref. | Ref. |
| Yes | 1(0.91,1.09) | 0.96(0.89,1.04) |
| **Difficulty in IADL** |  |  |
| No | Ref. | Ref. |
| Yes | 1.17*(1.09,1.27) | 1.1*(1.03,1.18) |
| **Psychological** |  |  |
| Low | Ref. | Ref. |
| Medium | 1.96*(1.82,2.12) | 1.9*(1.76,2.04) |
| High | 2.93*(2.7,3.18) | 3.04*(2.81,3.28) |
| **MPCE quintile** |  |  |
| Poorest | 1.17*(1.05,1.31) | 1.44*(1.3,1.6) |
| Poorer | 1.1(0.99,1.22) | 1.16*(1.05,1.29) |
| Middle | 1.07(0.97,1.19) | 1.13*(1.02,1.25) |
| Richer | 1.03(0.93,1.14) | 1.09(0.98,1.2) |
| Richest | Ref. | Ref. |
| **Religion** |  |  |
| Hindu | Ref. | Ref. |
| Muslim | 1.16*(1.05,1.28) | 1.17*(1.06,1.29) |
| Christian | 0.97(0.85,1.11) | 0.9(0.79,1.02) |
| Others | 0.96(0.82,1.13) | 0.94(0.81,1.09) |
| **Caste** |  |  |
| Scheduled Caste | 1.18*(1.06,1.31) | 1.22*(1.11,1.35) |
| Scheduled Tribe | 1.26*(1.12,1.41) | 1.12*(1,1.25) |
| Other Backward Class | 0.98(0.9,1.07) | 1(0.92,1.08) |
| Others | Ref. | Ref. |
| **Place of residence** |  |  |
| Rural | 1.06(0.98,1.14) | 1.16*(1.08,1.24) |
| Urban | Ref. | Ref. |
| **Region** |  |  |
| North | Ref. | Ref. |
| Central | 1(0.89,1.12) | 1.03(0.92,1.15) |
| East | 1.24*(1.12,1.38) | 1.46*(1.32,1.62) |
| Northeast | 0.96(0.84,1.11) | 1.13(0.99,1.28) |
| West | 0.47*(0.42,0.53) | 0.52*(0.47,0.59) |
| South | 1.15*(1.04,1.28) | 1.22*(1.1,1.35) |

*Ref: Reference; #: Interaction effect; UOR: Unadjusted odds ratio; AOR: Adjusted odds ratio; * if p<0.05; CI: Confidence interval*
